# Application of Outer Membrane Protein Omp25 in Serological Diagnosis of Brucellosis

**DOI:** 10.3390/idr18040086

**Published:** 2026-08-11

**Authors:** Qianhao Zhou, Dongqi Xiong, Xuandong Wang, Qingmei Zhu, Nadira Dolkun, Zureguli Tuxun, Zehao Zhang, Qian Wang, Xuejun Xiao

**Affiliations:** 1 Department of Pharmacology, School of Pharmacy, Xinjiang Medical University, No. 567 Shangde North Road, Shuimogou District, Urumqi 830017, China; 13547397577@163.com (Q.Z.); xiongdq2052@163.com (D.X.); wangxd114514@163.com (X.W.); z15001649407@163.com (Q.Z.); nadira_xyd@163.com (N.D.); zureguli0516@163.com (Z.T.); zhangze050711@163.com (Z.Z.); 2 School of Public Health, Xinjiang Medical University, Urumqi 830017, China; 3 Institute of Materia Medica, Xinjiang Medical University, Urumqi 830017, China

**Keywords:** Brucella, Omp25, outer membrane protein, serological diagnosis, diagnostic efficacy

## Abstract

Background: Brucellosis remains a widespread zoonotic disease where serodiagnosis is critical for control. Outer membrane protein 25 (Omp25) has emerged as a promising diagnostic antigen, yet the optimal antigen format for Omp25-based serological assays remains unclear. Methods: We performed a systematic review and meta-analysis of diagnostic accuracy studies following PRISMA-DTA 2020 guidelines. Four electronic databases were searched through July 16, 2026. Risk of bias was assessed using QUADAS-2. Diagnostic accuracy was synthesized using the bivariate binomial model, with subgroup analyses stratified by host species and antigen format. Results: Ten diagnostic accuracy studies comprising 23 study arms were included. Substantial heterogeneity was observed (I^2^ = 86.2% for sensitivity, 68.4% for specificity). In human serum, pooled sensitivity was 0.93 (95% CI: 0.89–0.96) and specificity was 0.97 (95% CI: 0.93–0.99). In ruminant serum, pooled sensitivity was 0.89 (95% CI: 0.81–0.94) and specificity was 0.95 (95% CI: 0.91–0.97). Multi-protein formats demonstrated narrower confidence intervals and more consistent diagnostic reliability than monomeric Omp25, despite a marginally lower pooled sensitivity point estimate. Single Omp25 exhibited the widest cross-species performance variation. Reference standard heterogeneity affected apparent specificity, with RBT + SAT dual standards yielding higher specificity than single RBT. Conclusions: Multi-protein antigen formats offer more consistent diagnostic reliability than monomeric Omp25 for brucellosis serodiagnosis, though performance varies significantly by host species. Clinical translation requires standardized antigen preparation protocols, uniform reference standards, and cautious species-specific application. Future research should prioritize larger, species-stratified, multi-center validation and prospective trials to establish Omp25-based DIVA capability.

## 1. Introduction

Brucellosis is a zoonotic disease caused by *Brucella* spp., which is prevalent worldwide and poses severe threats to animal husbandry production and public health. The human epidemic of brucellosis in China has shown a worsening trend since 2000 [1,2]. As a Gram-negative intracellular bacterium, Brucella comprises six classical species, among which *Brucella melitensis* is the most virulent and pathogenic to humans. Infected animals frequently suffer from reproductive disorders such as infertility and abortion, while humans infected via contact with sick animals or contaminated food present with clinical manifestations including persistent fever and central nervous system damage, and may even experience death or infertility in severe cases [3].

Serological diagnosis is a core component of brucellosis prevention and control, supporting epidemic surveillance, case confirmation, and epidemiological research. Brucella outer membrane proteins (OMPs) have become key diagnostic antigens due to their high immunogenicity and specificity, and indirect enzyme-linked immunosorbent assay (iELISA) based on these proteins has demonstrated favorable diagnostic performance in detecting sera from humans, cattle, and sheep [4,5]. However, traditional serological diagnosis has three major limitations: first, the use of whole bacteria or lipopolysaccharide (LPS) as antigens causes cross-reactivity with Gram-negative bacteria such as *Yersinia enterocolitica* O9 and *Escherichia coli* O157:H7, leading to misdiagnosis [6]; second, it cannot distinguish antibodies induced by vaccination from those generated by natural infection, hindering accurate epidemic assessment; third, the variable antigen reactivity to different host sera challenges the adaptability of diagnostic methods to diverse scenarios [7,8,9].

*Brucella* outer membrane protein 25 (Omp25) is a core member of the group 3 *Brucella* OMP family, which is highly conserved across all *Brucella* species [10]. As a major virulence factor released during host cell invasion, Omp25 activates the mitogen-activated protein kinase pathway, regulates the secretion of cytokines such as tumor necrosis factor α (TNF-α) and interleukin 1 (IL-1), and induces the production of specific antibodies in the host, enabling serological detection of infection status [11]. Bioinformatics analyses have confirmed that *Brucella melitensis* Omp25 contains 213 amino acids, with 1 CD4+ T-cell epitope, 3 CD8+ T-cell epitopes, and 3 B-cell dominant epitopes predicted [12,13,14]. The expression of Omp25 is directly regulated by the *Brucella* two-component regulatory system BvrR/BvrS, and its expression level is closely associated with the intracellular survival and virulence of the bacterium; thus, detection of Omp25-specific antibodies in serum can reflect the progression of infection [15,16].

Traditional diagnostic methods fail to distinguish natural infection, vaccination, and cross-infection due to the cross-reactivity of smooth LPS (S-LPS) with vaccine-induced antibodies and specific Gram-negative bacteria [17]. Additionally, these methods overlook the regulatory role of Omp25 in the synthesis of antigen-presenting molecules in dendritic cells (DCs), resulting in insufficient diagnostic sensitivity for samples with low antibody concentrations in the early stage of infection [18]. Current research on Omp25-based diagnosis is fragmented and lacks a unified standardized framework [19,20]. Therefore, this systematic review and meta-analysis was conducted with the following specific objectives: (i) to systematically evaluate the diagnostic accuracy of Omp25-based serological assays for brucellosis across different host species; (ii) to compare the diagnostic performance of monomeric Omp25, mixed-protein combinations, and multi-epitope fusion proteins; (iii) to identify sources of between-study heterogeneity, including host species, antigen format, and reference standard variability; and (iv) to provide evidence-based recommendations for the clinical translation and standardization of Omp25-based serodiagnosis. Clarifying the diagnostic value and application scenarios of Omp25 and related antigens is imperative to optimize the brucellosis prevention and control system.

## 2. Methods

This systematic review was conducted and reported in strict accordance with the Preferred Reporting Items for Systematic Reviews and Meta-Analyses for Diagnostic Test Accuracy (PRISMA-DTA) 2020 guidelines. The completed PRISMA 2020 checklist is provided in Supplementary Table S1. The study protocol was not registered in PROSPERO due to the exploratory nature of this diagnostic accuracy synthesis.

### 2.1. Databases and Search Strategy

We systematically searched PubMed, Web of Science Core Collection, China National Knowledge Infrastructure (CNKI), and Wanfang Data from their inception to March 15, 2026. All four databases were queried on July 16, 2026. No language restrictions were applied to avoid publication bias.

PubMed search formula: (“Brucella” [TIAB] OR “Brucellosis” [TIAB]) AND (“OMP25” [TIAB] OR “outer membrane protein” [TIAB]) AND (“diagnos*” [TIAB] OR “sensitivity” [TIAB] OR “specificity” [TIAB] OR “ROC curve” [TIAB]).

Web of Science search formula: TS = ((“Brucella” OR “Brucellosis”) AND (“OMP25” OR “outer membrane protein”) AND (“diagnos*” OR “sensitivity” OR “specificity” OR “ROC curve” OR “AUC”)) AND DT = (“Article”).

CNKI & Wanfang (searched in Chinese): (Subject: Brucella OR Subject: brucellosis OR Subject: undulant fever) AND (Subject: Omp25 OR Subject: outer membrane protein 25 OR Subject: outer membrane protein) AND (Subject: diagnosis OR Subject: serology OR Subject: ELISA OR Subject: sensitivity OR Subject: specificity OR Subject: ROC).

Duplicate studies were identified and removed using EndNote software (EndNote 2025). Two independent reviewers screened all records in duplicate; discrepancies were resolved by a third senior reviewer.

### 2.2. Inclusion and Exclusion Criteria

This systematic review was conducted in accordance with the PRISMA-DTA 2020 guidelines. The PICOS framework was defined as follows: Population (P) comprised individuals or animals with suspected or confirmed *Brucella* infection, including human patients, livestock (cattle, sheep, goats, dogs), and experimentally infected mice; Studies were required to include a minimum of 30 samples to ensure stable estimation of sensitivity and specificity. This threshold was pragmatically chosen rather than based on a formal power calculation. Intervention (I) consisted of serological assays using Omp25 as the core antigen across three formats, monomeric recombinant Omp25 protein, mixed-protein combinations containing Omp25, and Omp25-based multi-epitope fusion proteins; Reference standard (C) included conventional serological standards (Rose Bengal Plate Test (RBT), Standard Tube Agglutination Test (SAT), or RBT combined with SAT) and composite microbiological standards (bacterial isolation plus Polymerase Chain Reaction (PCR)); Outcomes (O) were diagnostic accuracy parameters, namely sensitivity, specificity, and diagnostic odds ratio (DOR) with 95% confidence intervals, with subgroup analyses stratified by host species and antigen format; Study design (S) encompassed diagnostic accuracy studies synthesized through systematic review and meta-analysis, with risk of bias assessed using the built-in QUADAS-2 module in RevMan software (Version 5.3) and quantitative pooling performed via the bivariate binomial model.

### 2.3. Data Extraction and Quality Assessment

**Figure 1:**
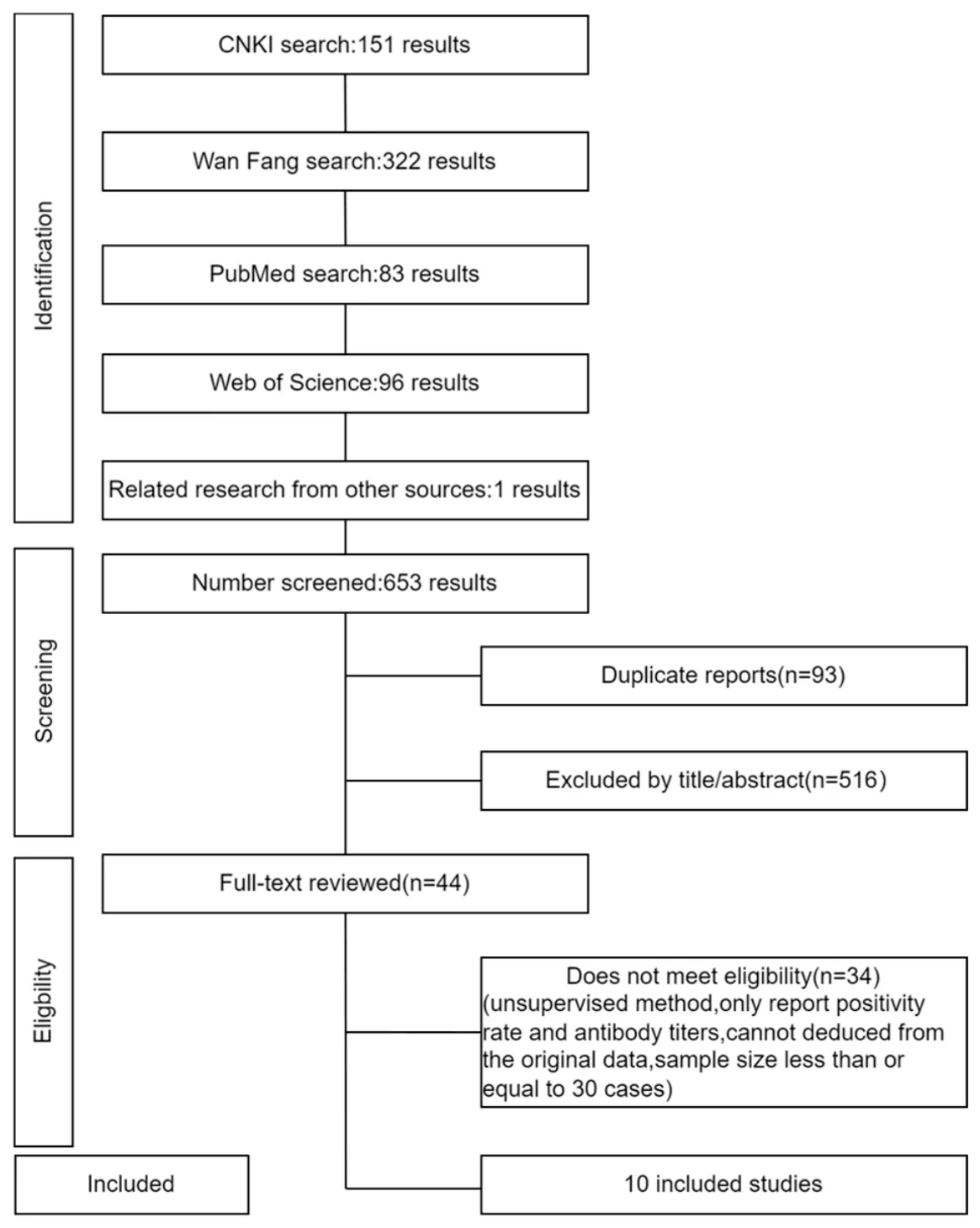
PRISMA flowchart for study screening and inclusion.

Data extraction and quality assessment were conducted in strict accordance with the PRISMA-DTA 2020 guidelines [21]. Two reviewers independently screened titles and abstracts, followed by full-text review, using a predefined eligibility checklist based on the PICOS framework. Discrepancies at any stage were resolved by consensus or, when necessary, arbitration by a third senior reviewer. The detailed workflow is illustrated in Figure 1.

A standardized data extraction form was used to collect: (i) study characteristics (first author, publication year); (ii) participant characteristics (host species, sample size); (iii) index test details (antigen format, ELISA platform, cut-off value and determination method); (iv) reference standard composition (RBT, SAT, RBT + SAT, or bacterial isolation plus PCR); and (v) 2 × 2 contingency table data (true positives, false positives, false negatives, true negatives) to enable direct calculation of sensitivity and specificity with 95% confidence intervals.

The Quality Assessment of Diagnostic Accuracy Studies 2 (QUADAS-2) tool [22] was independently applied by both reviewers to evaluate the risk of bias across four domains (patient selection, index test, reference standard, flow and timing). Each domain was rated as low, high, or unclear risk. Inter-reviewer disagreements were resolved by consensus discussion.

### 2.4. Statistical Analysis

Statistical analysis was conducted using Stata 18. True positives (TP), false positives (FP), false negatives (FN), and true negatives (TN) were extracted for quantitative synthesis; sensitivity and specificity with 95% confidence intervals (CIs) were calculated using the Wilson score method. The bivariate binomial model was used to jointly pool sensitivity and specificity and to perform subgroup analyses; the hierarchical summary ROC (HSROC) model was not selected because the small study number and sparse data rendered its additional parameters unstable. Forest plots were generated using the DerSimonian–Laird random-effects model for visual display only, whereas all pooled estimates were derived exclusively from the bivariate model. A summary ROC (SROC) curve was generated from the bivariate model estimates to visualize the summary operating point, 95% confidence region, and 95% prediction region. Heterogeneity was quantified using the I^2^ statistic, with I^2^ > 50% indicating substantial heterogeneity.

Several 2 × 2 tables contained zero false positives or false negatives. The bivariate model accommodates zero cells through built-in numerical estimation; a continuity correction of 0.5 was applied to variance calculations for forest plot display. Studies with zero-cell-related convergence failure were automatically excluded from bivariate estimation. In the RBT + SAT subgroup, four study arms were excluded due to zero cells in the 2 × 2 contingency tables, reducing the effective sample size from 11 to 7. Subgroup analyses were conducted by host species, reference standard, and antigen format. Animal studies were stratified into ruminants and non-ruminants; due to insufficient data (k = 3), bivariate meta-analysis was not performed for the non-ruminant subgroup.

Meta-regression was performed at the study level (*n* = 10) to examine the independent effects of host species and reference standard type on the diagnostic odds ratio (DOR). Meta-regression was performed with 10 studies and 2 covariates (host species and reference standard). This falls below the recommended threshold of ≥10 studies per covariate. Consequently, these analyses are likely underpowered and susceptible to overfitting, and should be regarded as purely exploratory. The estimates may be unstable and the wide confidence intervals reflect imprecision rather than evidence of absence. The primary purpose was to generate hypotheses about potential sources of heterogeneity, with the understanding that the limited statistical power may preclude detection of true effects and that the estimates may be unstable. One representative study arm per study was selected to satisfy the independence assumption. A continuity correction of 0.5 was applied to all cells prior to DOR calculation, and the Knapp-Hartung adjustment was used. Statistical significance was set at *p* < 0.05.

## 3. Results

### 3.1. Basic Characteristics of Included Studies

A total of 10 diagnostic accuracy studies were included in this research. Substantial between-study heterogeneity was observed for both sensitivity and specificity. The I^2^ value for sensitivity was 86.2% (*p* < 0.001) and the I^2^ value for specificity was 68.4% (*p* < 0.001), confirming significant heterogeneity across the included studies. Cut-off values were extracted as reported by original studies; no recalibration or standardization was performed because primary patient-level data were unavailable. This threshold variability is acknowledged as a source of heterogeneity.

Three main sources of heterogeneity were identified: (1) Host species differences: the sensitivity of Omp25 in bovine serum was approximately 62.77%, while it reached 100% in ovine serum, related to differential regulation of Omp25 expression across *Brucella* species; (2) Variability of reference standards: studies using a single RBT showed low specificity, while the RBT + SAT dual standard increased specificity to over 95%; (3) Variations in prokaryotic expression system parameters may contribute to heterogeneity. Standardized antigen preparation protocols and uniform reference standards are needed to improve methodological reliability.

### 3.2. Data Extraction from the Included Studies

**Figure 2:**
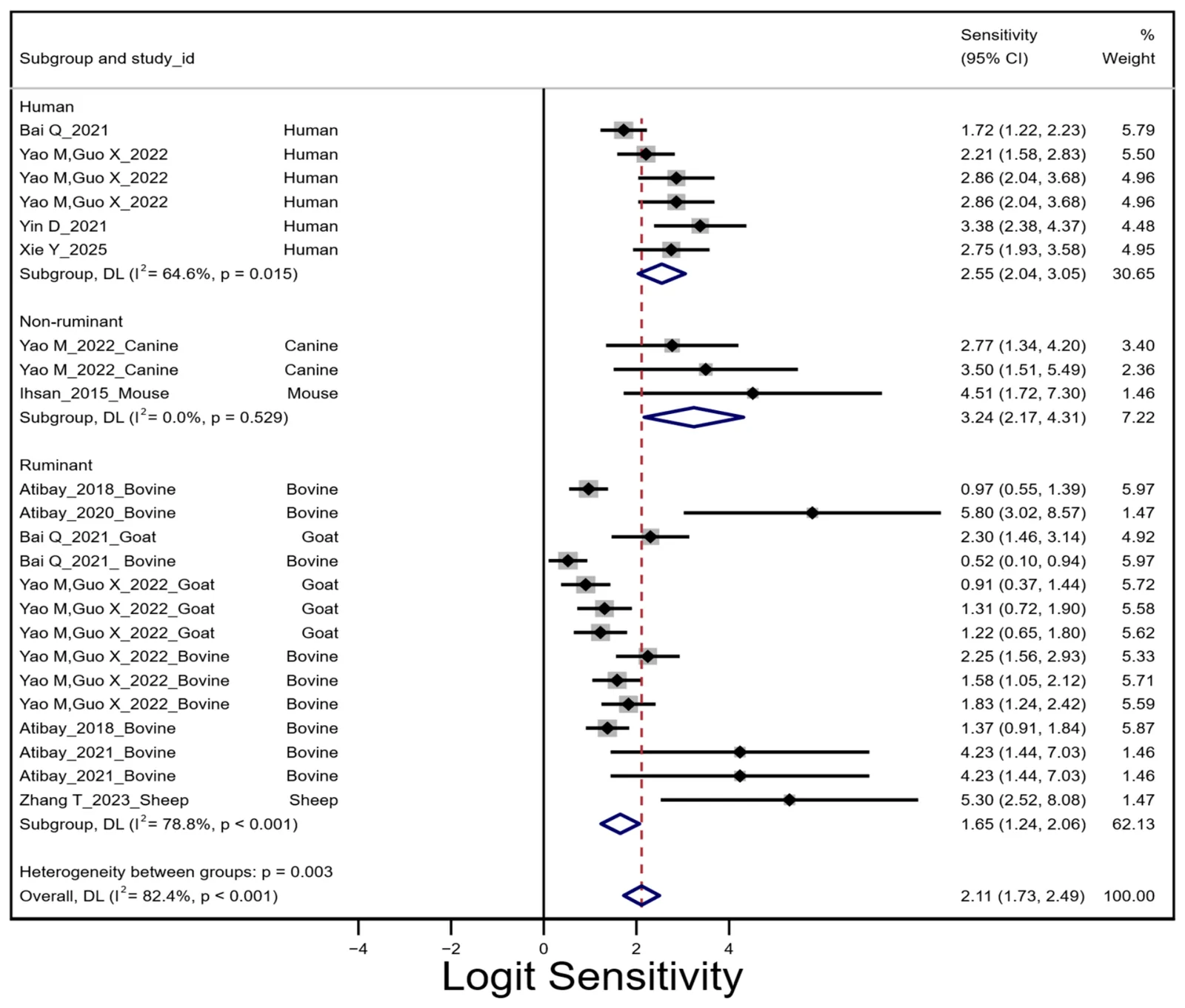
Forest plot of sensitivity by host species (Human, Ruminant, and Non-ruminant subgroups). All studies included in this forest plot are referenced in Table 1, Table 2, Table 3, Table 4, Table 5, Table 6 and Table 7 as [23,24,25,26,27,28,29,30,31,32]. The squares represent the point estimates of sensitivity for individual studies, and the horizontal lines indicate their 95% confidence intervals (CIs). The blue diamonds represent the pooled summary estimates for each subgroup and for all studies combined, with the width of each diamond indicating its respective 95% CI. The vertical solid line represents the line of no effect (logit sensitivity = 0), and the vertical dotted line represents the overall pooled estimate.

**Figure 3:**
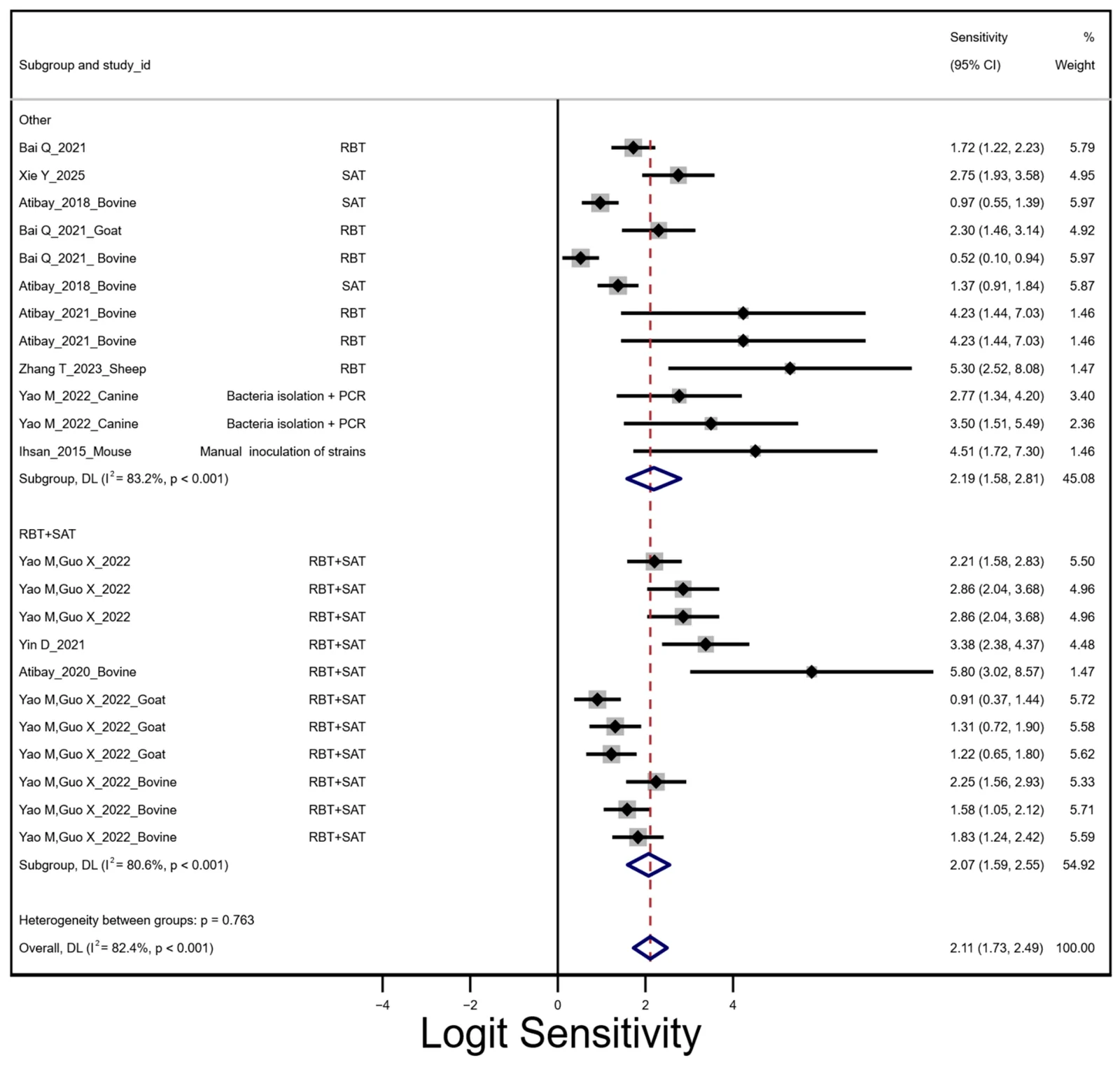
Forest plot of sensitivity by reference standard (RBT + SAT vs. Other reference standards). All studies included in this forest plot are referenced in Table 1, Table 2, Table 3, Table 4, Table 5, Table 6 and Table 7 as [23,24,25,26,27,28,29,30,31,32]. The squares represent the point estimates of sensitivity for individual studies, and the horizontal lines indicate their 95% confidence intervals (CIs). The blue diamonds represent the pooled summary estimates for each subgroup and for all studies combined, with the width of each diamond indicating its respective 95% CI. The vertical solid line represents the line of no effect (logit sensitivity = 0), and the vertical dotted line represents the overall pooled estimate.

Based on the 2 × 2 contingency data extracted from all eligible studies (Table 1, Table 2, Table 3, Table 4, Table 5, Table 6 and Table 7), the overall diagnostic accuracy of Omp25-based serological assays was synthesized using the bivariate binomial model. The forest plot of sensitivity stratified by host species (Figure 2) and the forest plot of sensitivity stratified by reference standard (Figure 3) were generated using the DerSimonian–Laird random-effects model for visual display only, whereas all pooled estimates were derived exclusively from the bivariate model. Specifically, the I^2^ statistic for sensitivity was 86.2% (*p* < 0.001) and for specificity was 68.4% (*p* < 0.001), confirming remarkable between-study heterogeneity across all analyses.

**Figure 4:**
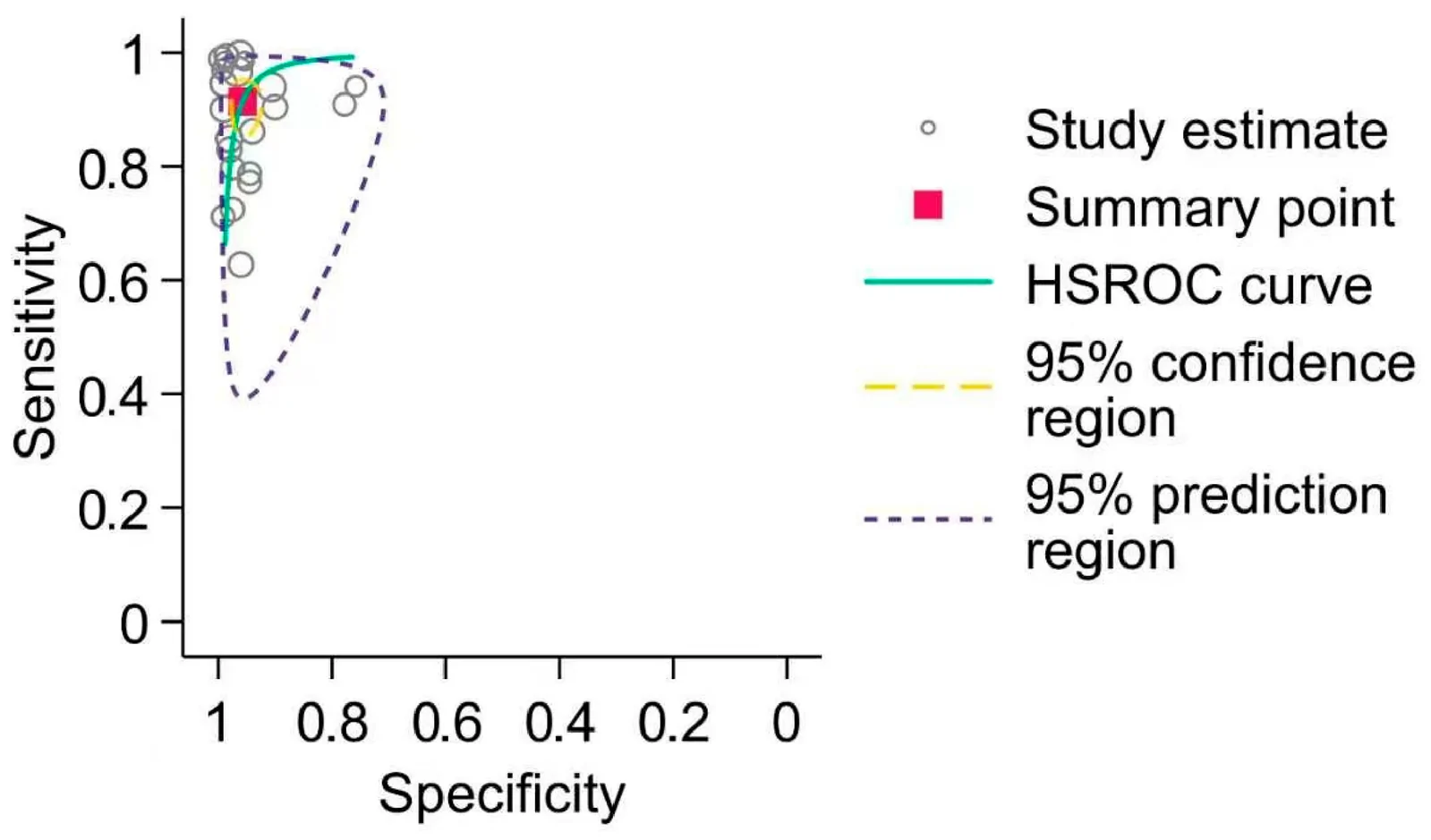
Summary receiver operating characteristic (SROC) curve of Omp25-based serological assays for brucellosis diagnosis.

A summary receiver operating characteristic (SROC) curve (Figure 4) derived from the bivariate model was generated to visualize the summary operating point, 95% confidence region, and 95% prediction region.

Subgroup analyses stratified by antigen format were further conducted using the bivariate binomial model to explore the sources of heterogeneity and compare diagnostic performance across subgroups (Table 8). Given the limited overall evidence base, mixed-protein combinations and multi-epitope fusion proteins were consolidated into a single multi-protein group for the antigen format comparison. This aggregation was necessary to ensure sufficient data density for stable bivariate estimation, as separate stratification would have yielded overly sparse strata with unstable confidence intervals. Furthermore, both formats share the common immunological strategy of expanding epitope coverage beyond monomeric Omp25, justifying their joint evaluation against the single-protein reference.

For the host species subgroup, ruminants were consolidated for bivariate analysis; non-ruminants were reported descriptively only because individual species were represented by sparse, mostly single-study data that precluded stable species-specific bivariate estimation. In human serum, the pooled sensitivity was 0.93 (95% CI: 0.89–0.96) and pooled specificity was 0.97 (95% CI: 0.93–0.99), with a diagnostic odds ratio (DOR) of 387.64 (95% CI: 170.47–881.44), a positive likelihood ratio (LR+) of 28.05 (95% CI: 13.09–60.10), and a negative likelihood ratio (LR−) of 0.072 (95% CI: 0.047–0.111). In ruminant serum, the pooled sensitivity was 0.89 (95% CI: 0.81–0.94), pooled specificity was 0.95 (95% CI: 0.91–0.97), DOR was 142.46 (95% CI: 64.95–312.51), LR+ was 16.67 (95% CI: 10.33–26.91), and LR− was 0.117 (95% CI: 0.065–0.211). Due to insufficient research data items (k = 3), bivariate meta-analysis was not performed for the non-ruminant subgroup; only descriptive statistics were reported (Table 2, Table 3, Table 4, Table 5 and Table 6).

Meta-regression was performed at the study level (*n* = 10) to examine the independent effects of host species and reference standard type on the diagnostic odds ratio (DOR). Meta-regression showed a marginal association between host species (human vs. ruminant) and diagnostic accuracy (coefficient = −1.985, 95% CI: −4.126 to 0.157; *p* = 0.065), with tau^2^ = 0.455, residual I^2^ = 25.28%, and adjusted R^2^ = 63.03%, suggesting that host species may explain a substantial proportion of between-study variance, although statistical significance was not reached given the small sample size. For reference standard type, meta-regression showed no significant association with diagnostic accuracy (coefficient = 0.886, 95% CI: −1.876 to 3.647; *p* = 0.481), with tau^2^ = 1.26, residual I^2^ = 44.81%, and adjusted R^2^ = −2.58%.

Subgroup analyses by reference standard were also conducted using the bivariate binomial model. Among studies employing the RBT + SAT dual reference standard (k = 7), the pooled sensitivity was 0.87 (95% CI: 0.76–0.94), pooled specificity was 0.95 (95% CI: 0.91–0.97), DOR was 116.64 (95% CI: 56.81–239.50), LR+ was 15.73 (95% CI: 9.58–25.85), and LR− was 0.135 (95% CI: 0.069–0.263). Among studies using other reference standards (k = 12), the pooled sensitivity was 0.93 (95% CI: 0.85–0.96), pooled specificity was 0.95 (95% CI: 0.90–0.98), DOR was 239.60 (95% CI: 79.61–721.11), LR+ was 19.01 (95% CI: 9.58–38.44), and LR− was 0.080 (95% CI: 0.039–0.160).

For the antigen format subgroup, the single Omp25 protein group showed a pooled sensitivity of 0.95 (95% CI: 0.75–0.99) and pooled specificity of 0.97 (95% CI: 0.82–0.99), while the multi-protein group (including mixed-protein combinations and multi-epitope fusion proteins) yielded a pooled sensitivity of 0.92 (95% CI: 0.86–0.95) and pooled specificity of 0.98 (95% CI: 0.95–0.99). These subgroup results demonstrated the stable and favorable diagnostic efficacy of Omp25-based serological assays, and further verified that host species and antigen formulation were key factors contributing to the observed between-study heterogeneity.

The non-ruminant subgroup comprised two canine study arms and one murine study arm, with sensitivities of 94.12%, 97.06%, and 100% and specificities of 75.81%, 100%, and 100%, respectively.

## 4. Discussion

### 4.1. Comparative Analysis of Omp25 Diagnostic Efficacy Among Different Application Formats

The diagnostic performance of Omp25-based assays varies markedly by antigen formulation. In human serum, monomeric recombinant Omp25 showed the lowest sensitivity (84.87%), whereas protein mixtures (Omp25 + Omp31 + BP26) reached 94.59%, and multi-epitope fusion proteins achieved the highest sensitivity (96.69%) (Table 2). Notably, the two multi-epitope studies in human serum reported sensitivities of 96.69% and 94.00%, respectively, with the latter exhibiting lower specificity (90.63%), suggesting study-level heterogeneity in multi-epitope performance. Pooled subgroup analysis by antigen format (Table 8) indicates that the multi-protein group demonstrated tighter confidence intervals than monomeric Omp25, reflecting lower heterogeneity and more consistent diagnostic reliability. Although pooled across-species analysis showed a slightly lower point estimate for multi-protein sensitivity than for monomeric Omp25—driven by high-sensitivity single-protein animal studies—the multi-protein group exhibited markedly narrower confidence intervals, indicating more consistent diagnostic reliability. The experimental mouse data are limited to a single study, so any inference about antigen performance in murine models remains preliminary. It should be emphasized that the included diagnostic accuracy studies did not directly investigate the molecular mechanisms underlying Omp25 immunogenicity. The following mechanistic interpretation draws on complementary structural and immunological evidence to provide a plausible biological rationale for the observed diagnostic patterns, but remains hypothetical pending direct experimental validation within diagnostic accuracy frameworks. This hierarchy reflects the antigenic complexity of natural *Brucella* infection. Outer membrane vesicles expose multiple OMPs eliciting polyclonal responses [33]; Omp25 and Omp31 act synergistically during invasion [34]. Omp25 suppresses IL-12 via NF-κB [35], dampening early antibodies. Wild-type *B. abortus* activates broader MAPK signaling [36] than recombinant monomers, and Omp25 harbors conformational epitopes lost in prokaryotic expression [37]. Inclusion bodies lack native folding [38], and the Omp25/Omp31 family maintains membrane stability [39]. Multi-epitope fusion proteins concatenate immunodominant regions [40], expanding coverage.

### 4.2. Cross-Species Variability in Omp25-Based Diagnostic Adaptability

Subgroup analysis by host species revealed a pooled sensitivity of 0.93 (95% CI: 0.89–0.96) and specificity of 0.97 (95% CI: 0.93–0.99) for human serum, and a pooled sensitivity of 0.89 (95% CI: 0.81–0.94) and specificity of 0.95 (95% CI: 0.91–0.97) for ruminant serum, with ruminant studies displaying wider confidence intervals. Meta-regression at the study level (*n* = 10) showed a marginal association between host species and diagnostic accuracy (coefficient = −1.985, 95% CI: −4.126 to 0.157; *p* = 0.065), although this finding was limited by small sample size and zero-cell inflation in several animal studies. In bovine serum, single Omp25 sensitivity ranged from 62.77% to 100%, while specificity was as low as 77.78% in goats and 75.81% in dogs. Most livestock species were represented by single or few studies, precluding stable species-specific bivariate estimation. Consequently, ruminants were consolidated for analysis, while non-ruminants were described separately to explore host-related heterogeneity rather than to derive robust species-specific estimates. Murine data were derived from a single experimental infection model and should be interpreted as exploratory. This variability arises from species-specific differences in Omp25 expression regulation and immunosuppressive magnitude. Castillo-Zeledon et al. [41] demonstrated that the response regulator BvrR binds directly to three DNA regulatory boxes in the Omp25 upstream region to activate transcription, and Altamirano-Silva et al. [42] established that this binding strictly depends on BvrR phosphorylation. Because intracellular triggers of BvrR phosphorylation differ across mammalian species, Omp25 expression levels and subsequent antibody kinetics vary among hosts. Murugan et al. [43] showed that Omp25 drives ubiquitin-proteasome degradation of TLR2, TLR4, TLR9, and adaptor proteins, dampening cytokine production and delaying antibody generation. Cui et al. [35] further demonstrated that Omp25 upregulates miR-155, miR-21-5p, and miR-23b via PD-1 signaling to inhibit IL-12, whereas Li et al. [44] showed that Omp25 promotes cGAS degradation and attenuates IFN-β across human, murine, porcine, bovine, and ovine cells. Ma et al. [45] observed that Omp25 induces microglial cells to secrete inflammatory cytokines through TLR4, revealing tissue-specific immune modulation. Additionally, Zhi et al. [46] identified Omp25D as an ArsR6-regulated virulence factor, indicating that compensatory expression among Omp25-family members alters the antigenic landscape in different hosts. Notably, multi-epitope fusion proteins raised canine specificity from 75.81% to 100% and sensitivity from 94.12% to 97.06% (Table 4), indicating that broadened epitope coverage stabilized detection in the evaluated species, suggesting potential for wider cross-species applicability pending further data.

To further disentangle the independent contributions of host species and reference standard heterogeneity, we performed meta-regression at the study level (*n* = 10). This analysis should be interpreted with substantial caution: three studies (Bulashev et al. 2020 [28], Zhang T et al. 2023 [30], Ihsan et al. 2015 [32]) contained zero-cell counts, and the required continuity correction yielded extreme DOR values that may have distorted effect estimates. Moreover, with only 10 studies and two covariates, the analysis falls below the recommended threshold of ≥10 studies per covariate, rendering it underpowered and susceptible to overfitting.

Host species showed a marginal association with diagnostic accuracy (coefficient = −1.985, 95% CI: −4.126 to 0.157; *p* = 0.065), with tau^2^ = 0.455, residual I^2^ = 25.28%, and adjusted R^2^ = 63.03%. The wide confidence interval reflects imprecision inherent to a small sample size rather than evidence of absence, and the residual I^2^ indicates that host species alone does not fully account for observed heterogeneity. Nevertheless, the substantial adjusted R^2^ suggests that species differences may explain a meaningful proportion of between-study variance, and the near-significant *p* value should not be interpreted as definitive evidence of no effect.

By contrast, reference standard type showed no significant independent effect (coefficient = 0.886, 95% CI: −1.876 to 3.647; *p* = 0.481), with tau^2^ = 1.26, residual I^2^ = 44.81%, and a negative adjusted R^2^ (−2.58%). This implies that the apparent specificity differences between single RBT and RBT + SAT observed in subgroup analyses are likely confounded by concurrent variation in antigen format or other unmeasured methodological factors rather than representing a true independent effect of reference standard composition.

Taken together, these meta-regression results reinforce our conservative interpretation: while host species emerges as a plausible source of heterogeneity, the limited study number and methodological constraints preclude definitive attribution; reference standard effects should be evaluated within the context of antigen format rather than in isolation, and the wide confidence intervals should be viewed as indicators of insufficient evidence rather than evidence of absence.

### 4.3. Impact of Reference Standards on Omp25 Diagnostic Efficacy

Reference standard heterogeneity represents a major methodological limitation across the included studies, with implications that extend beyond a mere acknowledgment. Among the ten diagnostic accuracy studies, reference standards varied substantially: human studies employed RBT alone, RBT combined with SAT, or SAT alone; animal studies utilized SAT, RBT, RBT + SAT, or bacterial isolation supplemented with PCR. This variability directly impacts the validity of cross-study comparisons.

The empirical data demonstrate measurable effects on apparent specificity: for example, Bai Q et al. [23] reported 77.78% specificity for single Omp25 in goat serum when RBT alone was applied, whereas Yao M, Guo X et al. [24] observed 94.44–100% specificity for protein-mixture formats in the same host using the RBT + SAT dual standard. Pooled bivariate analysis further quantified this effect: among studies employing the RBT + SAT dual reference standard (k = 7), the pooled specificity was 0.95 (95% CI: 0.91–0.97), whereas among studies using other reference standards (k = 12), the pooled specificity was 0.95 (95% CI: 0.90–0.98). Notably, the RBT + SAT group showed lower pooled sensitivity (0.87, 95% CI: 0.76–0.94) compared to the other-standards group (0.93, 95% CI: 0.85–0.96), likely reflecting the more stringent case definition of the dual standard, which reduced false positives but potentially increased false negatives. Meta-regression confirmed no significant independent effect of reference standard type on diagnostic accuracy (coefficient = 0.886, 95% CI: −1.876 to 3.647; *p* = 0.481), suggesting that the observed differences in subgroup analyses may be confounded by other sources of heterogeneity, such as antigen format or host species. Vatankhah et al. [47] evaluated an rOmp2b-based ELISA against RBT and SAT, underscoring that even when recombinant OMP assays are validated against standardized serological references, independent calibration remains necessary for each antigen. Li et al. [48] developed a DPO-PCR assay targeting the Omp25 gene with a detection limit of 5.3 × 10^1^ CFU/mL and no cross-reactivity with non-target bacteria, suggesting that molecular confirmation could complement serological standards. However, none of the included diagnostic accuracy studies integrated such composite microbiological–molecular protocols. Consequently, inconsistent reference standards introduce quantifiable bias, and direct cross-study comparison remains problematic without methodological standardization.

### 4.4. Differentiation of Natural Infection and Vaccination

None of the ten included diagnostic accuracy studies incorporated vaccinated control groups; therefore, any inference regarding Omp25-based differentiation of natural infection from vaccination (DIVA) extends beyond the primary evidence base. External vaccine studies corroborate this limitation: Mohammadi et al. [49] demonstrated that a chimeric OMP25–OMP31 antigen formulated in chitosan nanoparticles elicited robust IgG and Th1-biased responses (IgG2a/IgG1 ratio ~1.59) in BALB/c mice, with 2.16 log units of protection. Gupta et al. [50] similarly reported that divalent Omp25 + L7/L12 immunization induced significant anti-Omp25 IgG titers in mice. He et al. [51] identified Omp25 as a dominant immunogen in *Brucella abortus* A19 bacterial ghost vaccines, confirming that diverse vaccine platforms, including live attenuated, subunit, and ghost vaccines, consistently induce anti-Omp25 antibodies. Shi et al. [20] and Elrashedy et al. [16] identified Omp25 core discriminatory epitopes: B-cell epitopes 59–71 and 52–71, with antibody titers in natural infection significantly higher than those induced by vaccination (accuracy >92.3%). CTL epitopes 7–15 and 61–69 are presented by MHC I molecules, and T-cell epitope 11–25 stimulates CD4+ T-cell proliferation (proliferation index 2.87 ± 0.31), supporting the potential of Omp25 for DIVA diagnosis.

Because the current diagnostic studies measured performance against conventional infection-status standards only, they provide no direct evidence that Omp25-specific antibody titers differ systematically between naturally infected and vaccinated hosts. Claims regarding DIVA utility remain hypothetical until prospective trials compare Omp25 serological responses in vaccinated versus naturally infected cohorts using identical antigen formats and reference standards.

### 4.5. Clinical Translation and Standardization

Translating the current evidence into clinical or veterinary practice requires careful qualification given the small number of studies, substantial statistical heterogeneity, inconsistent reference standards, and fundamental biological differences across human, ruminant, and murine systems. Yang et al. [52] recently demonstrated that Omp25 binds the ER chaperone BiP to activate all three branches of the unfolded protein response (PERK, IRE1α, and ATF6), thereby promoting intracellular proliferation and NF-κB-driven inflammation. While this mechanism provides a molecular rationale for Omp25 immunogenicity, it does not establish diagnostic cut-offs, clinical algorithms, or species-specific thresholds. Although the bivariate binomial model was applied to account for the correlation between sensitivity and specificity and to permit simultaneous pooled estimation, the overall number of included studies remains small (*n* = 10). Subgroup stratification by antigen format further dilutes the effective sample size per stratum, and several 2 × 2 tables contained zero or near-zero event counts, which limits the stability of bivariate estimates. This is reflected in the comparatively wide confidence interval for single Omp25 sensitivity (0.75–0.99) versus the multi-protein group (0.86–0.95). Consequently, cross-species ranking of antigen formats based on these pooled point estimates should be interpreted cautiously. Until larger, species-stratified, multi-center validation studies with standardized reference standards and pre-registered protocols become available, clinical adoption of Omp25-based serodiagnosis should proceed cautiously, with format selection tailored to local prevalence and the target host species.

### 4.6. Limitations of This Study

This review has several limitations. First, the study protocol was not prospectively registered in PROSPERO. Although this review was conducted as an exploratory synthesis of emerging evidence, the absence of a pre-registered protocol increases the risk of post hoc decision-making bias, wherein analytical choices might be influenced by the data themselves. We have attempted to mitigate this by strictly adhering to the PRISMA-DTA 2020 guidelines [21] during both the conduct and reporting phases. Second, our search was restricted to peer-reviewed articles in four electronic databases, excluding gray literature such as conference abstracts, dissertations, and government reports. This exclusion may introduce publication bias, as studies with negative or non-significant results are less likely to be published in indexed journals, potentially leading to overestimation of pooled diagnostic accuracy. Future updates of this review should incorporate gray literature searches to ensure a more comprehensive and unbiased evidence base. Third, the evidence base remains small, and subgroup stratification diluted sample sizes within individual strata. Several 2 × 2 tables contained zero or near-zero event counts, limiting the stability of bivariate estimates, as reflected in wide confidence intervals for some subgroups—notably the single Omp25 protein group and the non-ruminant category, for which bivariate meta-analysis was not performed due to insufficient study numbers. Fourth, reference standards varied substantially across studies, introducing quantifiable bias and undermining cross-study comparability. The meta-regression analyses were conducted with 10 studies and two covariates, which falls below the recommended threshold of ≥10 studies per covariate. Consequently, these analyses were likely underpowered and susceptible to overfitting, potentially yielding unstable estimates; the wide confidence intervals should not be interpreted as evidence of absence, but rather as an indication that the available evidence is insufficient to draw firm conclusions. Fifth, human clinical samples, animal clinical samples, and experimental mouse models were analyzed together; these systems differ biologically in antibody kinetics and Omp25 expression regulation. Murine data were derived from a single study, rendering any cross-species inference exploratory. Finally, all included studies used ELISA-based serology without vaccinated control groups, providing no direct evidence for differentiating natural infection from vaccination.

## 5. Conclusions

Omp25 is a promising diagnostic antigen for brucellosis serodiagnosis, with efficacy substantially influenced by antigen format and host species. Multi-protein antigen formats demonstrate more consistent diagnostic reliability than monomeric Omp25, as reflected by narrower confidence intervals and lower heterogeneity, despite a marginally lower pooled sensitivity point estimate driven by high-performing single-protein animal studies. Single Omp25 exhibits the widest cross-species performance variation, limiting its universal applicability. Host species appears to be a major source of between-study heterogeneity, whereas reference standard type does not show an independent effect on diagnostic accuracy. The absence of vaccinated control cohorts in all included studies precludes definitive conclusions regarding differentiation of natural infection from vaccination. Clinical adoption should proceed cautiously with antigen format selection tailored to the target host species and local prevalence. Future research should prioritize standardized antigen preparation protocols, uniform reference standards, larger species-stratified multi-center validation, and prospective trials incorporating vaccinated controls to establish Omp25-based DIVA capability.

## Figures and Tables

**Table 1:** Diagnostic Information of Three Omp25 Antigen Formats in Human Serum.

Antigen Format	Literature	Year	Protein	Diagnostic Methods	Reference Standard	Detection Wavelength (nm)	Cut-Off	AUC and Its 95% Confidence Interval
Single Omp25	Bai Q et al. [23]	2021	Omp25	iELISA	RBT	450	OD ≥ 0.69	NA
Protein mixture	Yao M, Guo X et al. [24]	2022	Omp25, Omp31	iELISA	RBT + SAT	450	OD ≥ 0.4739	NA
			Omp25, Omp31, BP26	iELISA	RBT + SAT	450	OD ≥ 0.4756	0.9976 (0.9890–1.001)
			Omp25, Omp31, BP26, Omp10, Omp19, Omp16	iELISA	RBT + SAT	450	OD ≥ 0.4261	NA
Multi-epitope Fusion Protein	Yin D et al. [25]	2021	Omp25, Omp31, BP26, Omp2b, Omp16	iELISA	RBT + SAT	450	OD ≥ 0.47	0.9877 (0.9758, 0.9996)
	Xie Y et al. [26]	2025	Omp25, BP26, Omp10, Omp2a, Omp31, Omp16, Omp2b	iELISA	SAT	450	OD ≥ 0.2774	0.9373 (0.9373, 0.9874)

iELISA: indirect enzyme-linked immunosorbent assay; RBT: Rose Bengal Plate Test; SAT: Standard Tube Agglutination Test; OD: optical density; AUC: area under the curve; CI: confidence interval; NA: Not Available.

**Table 2:** Diagnostic Efficacy Data of Three Omp25 Antigen Formats in Human Serum.

Antigen Format	Literature	Year	Protein	TP	FP	FN	TN	*n*	Sensitivity	Specificity
Single Omp25	Bai Q et al. [23]	2021	Omp25	101	1	18	49	169	84.87% (0.773,0.905)	98.00% (0.897, 0.998)
Protein mixture	Yao M, Guo X et al. [24]	2022	Omp25, Omp31	100	0	11	50	161	90.09% (0.829,0.950)	100.00% (0.929, 1.000)
			Omp25, Omp31, BP26	105	0	6	50	161	94.59% (0.885,0.979)	100.00% (0.929, 1.000)
			Omp25, Omp31, BP26, Omp10, Omp19, Omp16	105	0	6	50	161	94.59% (0.885,0.979)	100.00% (0.929, 1.000)
Multi-epitope Fusion Protein	Yin D et al. [25]	2021	Omp25, Omp31, BP26, Omp2b, Omp16	117	3	4	87	211	96.69% (0.918, 0.991)	96.67% (0.905, 0.993)
	Xie Y et al. [26]	2025	Omp25, BP26, Omp10, Omp2a, Omp31, Omp16, Omp2b	94	9	6	87	196	94.00% (0.875,0.976)	90.63% (0.829, 0.956)

TP: true positives; FP: false positives; FN: false negatives; TN: true negatives; *n*: total sample size; CI: confidence interval.

**Table 3:** Diagnostic Information of Three Omp25 Antigen Formats in Animal Serum.

Antigen Format	Literature	Year	Sample Type	Protein	Diagnostic Methods	Reference Standard	Detection Wavelength (nm)	Cut-Off	AUC and Its 95% Confidence Interval
Single Omp25	Aitbay et al. [27]	2018	Bovine serum	Omp25	iELISA	SAT	492	OD ≥ 0.156	NA
	Bulashev et al. [28]	2020	Bovine serum	Omp25	iELISA	RBT + SAT	490	NA	NA
	Bai Q et al. [23]	2021	Goat serum	Omp25	iELISA	RBT	450	OD ≥ 0.4729	NA
			Bovine serum	Omp25	iELISA	RBT	450	OD ≥ 0.7478	NA
	Yao M et al. [29]	2022	Canine serum	Omp25	iELISA	Bacteria isolation + PCR	450	OD ≥ 0.2165	0.9099 (0.8505, 0.9692)
	Zhang T et al. [30]	2023	Sheep serum	Omp25	iELISA	RBT	450	NA	NA
Protein mixture	Yao M, Guo X et al. [24]	2022	Goat serum	Omp25, Omp31	iELISA	RBT + SAT	450	OD ≥ 0.7119	NA
			Goat serum	Omp25, Omp31, BP26	iELISA	RBT + SAT	450	OD ≥ 0.628	NA
			Goat serum	Omp25, Omp31, BP26, Omp10, Omp19, Omp16	iELISA	RBT + SAT	450	OD ≥ 0.6724	NA
			Bovine serum	Omp25, Omp31	iELISA	RBT + SAT	450	OD ≥ 0.47	NA
			Bovine serum	Omp25, Omp31, BP26	iELISA	RBT + SAT	450	OD ≥ 0.6478	NA
			Bovine serum	Omp25, Omp31, BP26, Omp10, Omp19, Omp16	iELISA	RBT + SAT	450	OD ≥ 0.5398	NA
Multi-epitope Fusion Protein	Aitbay et al. [27]	2018	Bovine serum	Omp25, Omp31	iELISA	SAT	492	OD ≥ 0.120	NA
	Bulashev et al. [31]	2021	Bovine serum	Omp25, Omp31	iELISA	RBT	492	NA	NA
			Bovine serum	Omp25, Omp19	iELISA	RBT	492	NA	NA
	Yao M et al. [29]	2022	Canine serum	Omp25, Omp31, BP26, Omp2b, Omp16	iELISA	Bacteria isolation + PCR	450	OD ≥ 0.4855	0.9991 (0.9965, 1.002)

iELISA: indirect enzyme-linked immunosorbent assay; RBT: Rose Bengal Plate Test; SAT: Standard Tube Agglutination Test; PCR: Polymerase Chain Reaction; OD: optical density; AUC: area under the curve; CI: confidence interval; NA: Not Available.

**Table 4:** Diagnostic Efficacy Data of Three Omp25 Antigen Formats in Animal Serum.

Antigen Format	Literature	Year	Sample Type	Protein	TP	FP	FN	TN	*n*	Sensitivity	Specificity
Single	Aitbay et al. [27]	2018	Bovine serum	Omp25	79	0	30	19	128	72.48% (0.635, 0.800)	100.00% (0.832,1.000)
Omp25	Bulashev et al. [28]	2020	Bovine serum	Omp25	164	0	0	12	176	100.00% (0.979, 1.000)	100.00% (0.735,1.000)
	Bai Q et al. [23]	2021	Goat serum	Omp25	60	12	6	42	120	90.91% (0.815,0.962)	77.78% (0.647,0.875)
			Bovine serum	Omp25	59	2	35	48	144	62.77% (0.529,0.718)	96.00% (0.865,0.992)
	Yao M et al. [29]	2022	Canine serum	Omp25	32	15	2	47	96	94.12% (0.802,0.990)	75.81% (0.644,0.849)
	Zhang T et al. [30]	2023	Sheep serum	Omp25	100	0	0	33	133	100.00% (0.963,1.000)	100.00% (0.892,1.000)
Protein mixture	Yao M, Guo X et al. [24]	2022	Goat serum	Omp25, Omp31	47	0	19	54	120	71.21% (0.599,0.808)	100.00% (0.937, 1.000)
			Goat serum	Omp25, Omp31, BP26	52	3	14	51	120	78.79% (0.675,0.874)	94.44% (0.849, 0.983)
			Goat serum	Omp25, Omp31, BP26, Omp10, Omp19, Omp16	51	3	15	51	120	77.27% (0.658,0.862)	94.44% (0.849,0.983)
			Bovine serum	Omp25, Omp31	85	5	9	45	144	90.43% (0.827,0.953)	90.00% (0.782,0.967)
			Bovine serum	Omp25, Omp31, BP26	78	1	16	49	144	82.98% (0.736,0.900)	98.00% (0.895,0.995)
			Bovine serum	Omp25, Omp31, BP26, Omp10, Omp19, Omp16	81	3	13	47	144	86.17% (0.775,0.923)	94.00% (0.835,0.988)
Multi-epitope Fusion Protein	Aitbay et al. [27]	2018	Bovine serum	Omp25, Omp31	87	0	22	19	128	79.82% (0.708,0.869)	100.00% (0.824,1.000)
	Bulashev et al. [31]	2021	Bovine serum	Omp25, Omp31	34	0	0	43	77	100.00% (0.897,1.000)	100.00% (0.918,1.000)
			Bovine serum	Omp25, Omp19	34	2	0	41	77	100.00% (0.897,1.000)	95.35% (0.842,0.994)
	Yao M et al. [29]	2022	Canine serum	Omp25, Omp31, BP26, Omp2b, Omp16	33	0	1	62	96	97.06% (0.847,0.999)	100.00% (0.945,1.000)

TP: true positives; FP: false positives; FN: false negatives; TN: true negatives; *n*: total sample size; CI: confidence interval.

**Table 5:** Diagnostic Information of Omp25-Based Antigen Formats in Experimental Mouse Serum.

Antigen Format	Literature	Year	Protein	Reference Standard	Detection Wavelength (nm)	Cut-Off	AUC and Its 95% Confidence Interval
Protein mixture	Ihsan et al. [32]	2015	Omp25, Omp28, Omp31	Manual inoculation of strains	450	PP ≥ 43.30%	NA

Note: PP refers to Percentage of Positivity, a relative quantitative indicator for rOMPs I-ELISA. Calculation formula: PP = [(Optical Density (OD) value of test sample—OD value of negative control)/(OD value of positive control—OD value of negative control)] × 100%. The cut-off value for *Brucella* infection was determined by ROC analysis; NA: Not Available.

**Table 6:** Diagnostic Efficacy Data of Omp25-Based Antigen Formats in Experimental Mouse Serum.

Antigen Format	Literature	Year	Protein	TP	FP	FN	TN	*n*	Sensitivity	Specificity
Protein mixture	Ihsan et al. [32]	2015	Omp25, Omp28, Omp31	45	0	0	90	135	100.00% (0.921,1.000)	100.00% (0.960,1.000)

TP: true positives; FP: false positives; FN: false negatives; TN: true negatives; *n*: total sample size; CI: confidence interval.

**Table 7:** Risk of Bias Assessment Results of Included Studies (QUADAS-2 tool).

Literature	A. Risk of Bias	B. Index Test	C. Reference Standard	D. Flow and Timing
Bai Q et al. 2021 [23]	Unclear	Low	Unclear	Low
Yao M, Guo X et al. 2022 [24]	Unclear	Low	High	Low
Yin D et al. 2021 [25]	High	Unclear	Unclear	Low
Xie Y et al. 2025 [26]	Low	Low	Low	Low
Aitbay et al. 2018 [27]	Low	Low	Unclear	Low
Bulashev et al. 2020 [28]	High	Low	Low	Low
Yao M et al. 2022 [29]	Unclear	Low	Low	Low
Zhang T et al. 2023 [30]	Unclear	Low	Unclear	High
Bulashev et al. 2021 [31]	Unclear	Low	High	Low
Ihsan et al. 2015 [32]	Low	unclear	High	low

QUADAS-2: Quality Assessment of Diagnostic Accuracy Studies 2.

**Table 8:** Pooled Diagnostic Accuracy of Omp25-Based Assays Stratified by Antigen Format.

Group	Number of Study Arms	Pooled Sensitivity (95% CI)	Pooled Specificity (95% CI)	Diagnostic Odds Ratio (DOR) (95% CI)
Single Omp25	9	0.95 (0.75–0.99)	0.97 (0.82–0.99)	613.73 (29.33–12,845.60)
Multi-protein Group	14	0.92 (0.86–0.95)	0.98 (0.95–0.99)	603.08 (185.07–1965.19)

Note: Multi-protein Group includes mixed-protein combinations and multi-epitope fusion proteins. CI: confidence interval; DOR: diagnostic odds ratio.

## Data Availability

The data supporting the findings of this study are derived from published articles and are available within the reference list of this manuscript. Extracted 2 × 2 contingency tables and analysis scripts are available from the corresponding author upon reasonable request.

## Supplementary Materials

The following supporting information can be downloaded at https://www.mdpi.com/article/10.3390/idr18040086/s1. Table S1: PRISMA 2020 Checklist.

## Abbreviations

ELISA	Enzyme-Linked Immunosorbent Assay
Omp	Outer membrane protein
BvrR/BvrS	Brucella virulence response regulator/sensor kinase
ROC	Receiver Operating Characteristic
AUC	Area under the curve
PCR	Polymerase Chain Reaction
RBT	Rose Bengal Plate Test
SAT	Standard Tube Agglutination Test
CI	Confidence interval
TP	True positives
FN	False negatives
TN	True negatives
FP	False positives
QUADAS-2	Quality Assessment of Diagnostic Accuracy Studies 2
OD	Optical Density
BP	Brucella periplasmic
TLR	Toll-like receptor
